# Impeded Immunity? Reduced Tuberculosis Vaccine Response with Exposure to Environmental Chemicals

**DOI:** 10.1289/ehp.124-A114

**Published:** 2016-06-01

**Authors:** Lindsey Konkel

**Affiliations:** Lindsey Konkel is a New Jersey–based journalist who reports on science, health, and the environment.

There is some evidence that early-life exposures to polychlorinated biphenyls (PCBs) and other persistent environmental chemicals can alter the developing immune system and may be associated with diminished effectiveness for certain vaccines.^1^ ^,^ ^2^ This could have serious implications for parts of the world where diseases that are preventable with vaccines remain a major public health threat.^3^ In this issue of *EHP*, researchers present new evidence that two persistent organic pollutants are associated with a lower antibody response to the tuberculosis vaccine, which could potentially lower resistance to infection.^4^

“Our findings show that environmental chemicals may be playing a role in immune disruption—in this case the suppression of immune response to a vaccine,” says lead author Todd Jusko, an epidemiologist at the University of Rochester in New York.

**Figure:**
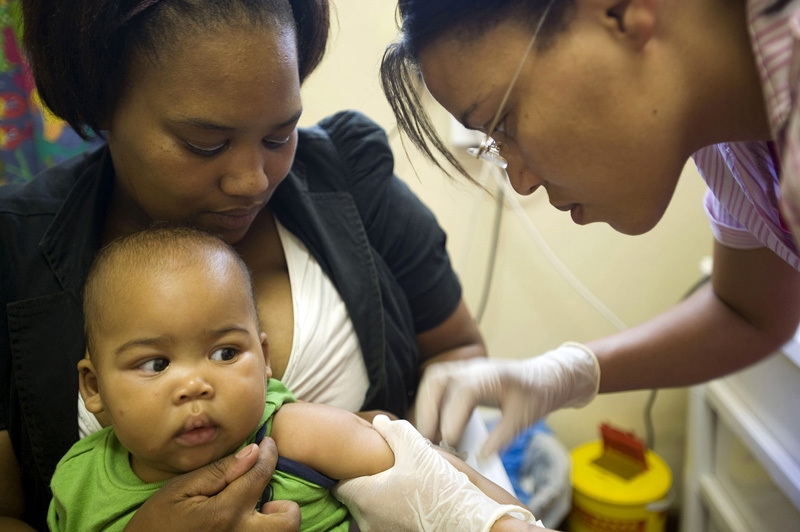
BCG, the most widely used vaccine in the world, is routinely given to babies in areas with high rates of tuberculosis. © Rodger Bosch/AFP/Getty Images

Although not routinely administered in the United States, the tuberculosis vaccine, called bacille Calmette-Guérin (BCG), is the most widely given vaccine in the world.^5^ BCG is administered shortly after birth, and in young children it has substantially reduced the risk of severe forms of tuberculosis in which the disease spreads beyond the lungs.^6^ But for unknown reasons the vaccine confers poor protection against pulmonary tuberculosis in older children and adults.^7^ Early-life environmental exposures may be an overlooked factor in the maintenance of immune protection over time, write the study authors.^4^

The researchers analyzed the blood of approximately 500 mother–infant pairs in eastern Slovakia for PCB-153 and for *p,p´-DDE-*DDE (a metabolite of the insecticide DDT). Their goal was to determine whether prenatal and early postnatal exposures to these chemicals were associated with reduced infant response to BCG at age 6 months. Both are considered chemicals of concern by the U.S. Environmental Protection Agency for their ability to persist in the environment and accumulate in the human body over time.^8^

The researchers found that infants with the highest blood levels of PCB-153 had an average 37% lower level of BCG-specific immunoglobin (Ig) G and A than infants with the lowest PCB exposures. Associations with the highest blood levels of *p,p´-*DDE were very similar to those for PCB-153. They also found that infants with the highest levels of both PCB-153 and *p,p´-*DDE had an average 85% lower level of IgA and 94% lower level of IgG, compared with infants with the lowest levels of both chemicals. Levels of PCB-153 and *p,p´-*DDE in maternal and cord blood samples collected at birth were not associated with infant antibody levels, suggesting that early infancy may be a critical period for environmental exposures to disrupt the developing immune system.^4^

While the study showed lower antibody levels in highly exposed children, indicating a weakened response, it’s unclear what these findings mean for the efficacy of the BCG vaccine. Experts aren’t sure at what level the vaccine stops protecting people from tuberculosis.

It’s also unclear whether PCB-153 and *p,p´-*DDE actually caused the antibody reductions. *p,p´-*DDE isn’t known to be particularly immunotoxic, points out Philippe Grandjean, an epidemiologist at the Harvard School of Public Health. Perhaps *p,p´-*DDE and PCB-153 are not the causative agents but rather markers for other chemicals in the same environment that are more immunotoxic, says Grandjean, who was not involved in the study.

The mechanisms by which environmental chemicals may suppress the immune system and diminish vaccine response are poorly understood. In a previous study with the Slovakian cohort, Jusko and colleagues found that infants with higher PCB exposures had a lower thymus volume.^9^ The thymus is an organ in the chest where specialized immune system cells called T-cells mature.

“Antibody production almost always is T-cell dependent,” says David Sherr, an immunotoxicologist at Boston University, who was not involved in the study. “The immune system is a complex and adaptive system. [Findings such as these] may just be scratching the surface.”

Jusko says more research is needed to address the pathways through which chemicals such as PCBs may suppress antibody response. “The immune system is a mediator of so many health outcomes,” says Jusko, “yet immune disruption by environmental chemicals is a pretty under-researched area of child development.”
